# Associations of pulmonary and extrapulmonary computed tomographic manifestations with impaired physical activity in symptomatic patients with chronic obstructive pulmonary disease

**DOI:** 10.1038/s41598-022-09554-6

**Published:** 2022-04-04

**Authors:** Yoko Hamakawa, Naoya Tanabe, Hiroshi Shima, Kunihiko Terada, Yusuke Shiraishi, Tomoki Maetani, Takeshi Kubo, Satoshi Kozawa, Koji Koizumi, Masashi Kanezaki, Kaoruko Shimizu, Tsuyoshi Oguma, Atsuyasu Sato, Susumu Sato, Toyohiro Hirai

**Affiliations:** 1 Department of Respiratory Medicine, Graduate School of Medicine, Kyoto University, 54 Kawahara-cho, Shogoin, Sakyo-ku, Kyoto, 606–8507 Japan; 2 Terada Clinic, Respiratory Medicine and General Practice, Himeji, Hyogo Japan; 3 Department of Diagnostic Imaging and Nuclear Medicine, Graduate School of Medicine, Kyoto University, Kyoto, Japan; 4 Division of Clinical Radiology Service, Kyoto University Hospital, Kyoto, Japan; 5 Department of Physical Therapy, School of Health Sciences, Tokyo International University, Kawagoe, Saitama Japan; 6 Department of Respiratory Medicine, Faculty of Medicine, Hokkaido University, Sapporo, Japan

**Keywords:** Chronic obstructive pulmonary disease, X-ray tomography

## Abstract

In patients with chronic obstructive pulmonary disease (COPD), emphysema, airway disease, and extrapulmonary comorbidities may cause various symptoms and impair physical activity. To investigate the relative associations of pulmonary and extrapulmonary manifestations with physical activity in symptomatic patients, this study enrolled 193 patients with COPD who underwent chest inspiratory/expiratory CT and completed COPD assessment test (CAT) and the Life-Space Assessment (LSA) questionnaires to evaluate symptom and physical activity. In symptomatic patients (CAT ≥ 10, n = 100), emphysema on inspiratory CT and air-trapping on expiratory CT were more severe and height-adjusted cross-sectional areas of pectoralis muscles (PM index) and adjacent subcutaneous adipose tissue (SAT index) on inspiratory CT were smaller in those with impaired physical activity (LSA < 60) than those without. In contrast, these findings were not observed in less symptomatic patients (CAT < 10). In multivariable analyses of the symptomatic patients, severe air-trapping and lower PM index and SAT index, but not CT-measured thoracic vertebrae bone density and coronary artery calcification, were associated with impaired physical activity. These suggest that increased air-trapping and decreased skeletal muscle and subcutaneous adipose tissue quantity are independently associated with impaired physical activity in symptomatic patients with COPD.

## Introduction

Chronic obstructive pulmonary disease (COPD) is characterized by airflow limitation induced by a combination of airway disease and emphysema in the lungs^1^. COPD is also characterized by extrapulmonary comorbidities such as muscle wasting, underweight, osteoporosis, and cardiovascular disease^2,3^. These pulmonary and extrapulmonary manifestations may cause physical inactivity and sedentary lifestyle^4,5^, leading to poor prognosis in patients with COPD^6,7^. However, due to the complex mixtures of the manifestations in and outside the lungs, determinants of decreased physical activity remain not fully understood and no personalized program to increase physical activity is currently available.

Airflow limitation induces air-trapping on expiration and lung hyperinflation that increase exertional dyspnea and daily symptoms^8^. While studies have shown associations of symptoms with physical inactivity^9,10^, the associations are modest. Indeed, symptomatic relief induced by bronchodilators and lung volume reductions in interventional studies is not always correlated with an increased physical activity^11,12^. Moreover, patients with COPD tend to avoid symptoms by decreasing physical activity^13^ and might be classified as those with less symptom and impaired physical activity. The discrepancy between symptoms and physical activity could also be accounted for by the extrapulmonary manifestations such as skeletal muscle loss and reduced bone mineral density, which have been shown to be associated with impaired physical activity^5,14,15^. Collectively, these findings suggest that factors associated with physical activity should be explored in symptomatic and less symptomatic patients, separately, by focusing on both the pulmonary and extrapulmonary manifestations.

Chest inspiratory and expiratory computed tomography allows simultaneously quantifying emphysema, airway disease, and air-trapping in the lungs^16^, as well as pectoralis muscle, erector spinae muscle, subcutaneous adipose tissue, bone mineral density (BMD) on thoracic vertebrae and coronary artery calcification outside the lungs^17–23^. Waschki et al. showed that emphysema severity on CT was associated with impaired physical activity^4^, whereas Tanimura et al. showed that a reduction in both pectoralis and erector spinae muscles was associated with a lower daily step count^19^. However, little is known about relative associations of the pulmonary and extrapulmonary CT findings with impaired physical activity in patients with COPD.

It was hypothesized that the lung pathophysiology and extrapulmonary manifestations are independently associated with impaired physical activity in patients with symptomatic COPD, but not those with less symptomatic COPD. Therefore, this study categorized patients with COPD based on symptom and physical inactivity using two questionnaires; COPD assessment test (CAT)^1^ and the Life-Space Assessment (LSA), which is easily performed to estimate physical activity by evaluating the extent of social isolation and sedentary lifestyle^24^. Then, the study aimed to compare the pulmonary and extrapulmonary CT findings of symptomatic and physically inactive patients to those of the remaining. Furthermore, the study constructed multivariable models to test whether the pulmonary and extrapulmonary CT findings could be independently associated with impaired physical activity in symptomatic patients.

## Results

### Patients’ characteristics

**Figure 1:**
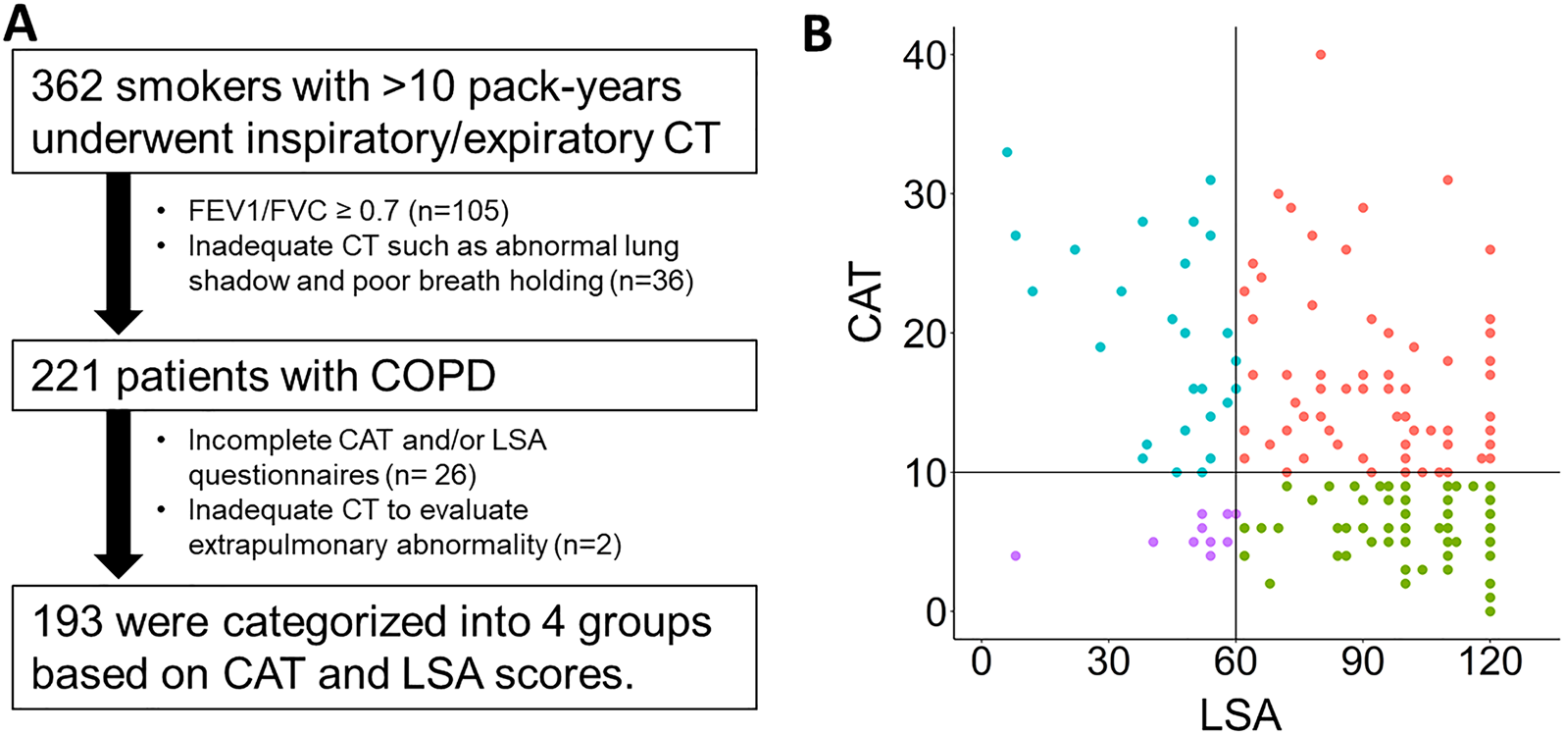
Patients’ flow and the distribution of COPD assessment test and Life-Space Assessment scores. Based on COPD assessment test (CAT) score of 10 and Life-Space Assessment (LSA) score of 60, patients were divided into 4 groups: (1) those with low CAT and high LSA (less symptomatic and physically active, n = 82), (2) low CAT and low LSA (less symptomatic and physically inactive, n = 11), (3) high CAT and high LSA (symptomatic and physically active, n = 73), (4) high CAT and low LSA (symptomatic and physically inactive, n = 27).

As shown in Fig. 1, of 362 smokers initially evaluated, 221 met the diagnostic criteria of COPD, but 26 were excluded because of incomplete CAT and/or LSA questionnaires and 2 were excluded because of inadequate CT quality. Total 193 patients with COPD were included for the present analyses and their demographics are shown in Table 1. As shown in Fig. 1B, based on CAT score of 10 and LSA score of 60, patients were divided into 4 groups: (1) those with low CAT and high LSA (less symptomatic and physically active, n = 82), (2) low CAT and low LSA (less symptomatic and physically inactive, n = 11), (3) high CAT and high LSA (symptomatic and physically active, n = 73), (4) high CAT and low LSA (symptomatic and physically inactive, n = 27).

**Table 1:** Patients’ Characteristics.

N	193
Age, years	72.5 ± 7.7
Male, n (%)	180 (93%)
Current smokers, n (%)	44 (23%)
Pack-years	60.2 ± 30.9
Height, cm	164.7 ± 7.1
BMI, kg/m^2^	23.2 ±
BMI ≥ 30 kg/m^2^, n (%)	6 (3%)
FEV_1_, % predicted	62.6 ± 22.6
FVC, % predicted	89.3 ± 22.0
FEV_1_ / FVC	0.53 ± 0.12
No. exacerbations in a previous year, 0/1/ ≥ 2	148/37/8
mMRC, 0/1/2/3/4	74 / 74 / 27 / 12 / 6
CAT ≥ 10, n (%)	100 (52%)
LSA ≤ 60, n (%)	38 (20%)
GOLD, 1/2/3/4	43/95/36/19
ABCD category, A / B / C / D	87/98/0/8

Data are expressed as mean ± SD and n (%). BMI = body mass index, Exacerbation in a past year = a history of at least one exacerbation in the previous one year, mMRC = modified MRC dyspnea scale, FEV_1_ = forced expiratory volume in 1 s, CAT = COPD assessment test, LSA = life space assessment.

### Clinical physiological features in symptomatic and physically inactive patients

As shown in Table 2, age, sex, smoking pack-years, height, number of exacerbations in a previous year, long-acting beta agonist (LABA) use, and inhaled corticosteroid (ICS) use did not differ between the 4 groups. The symptomatic and physically inactive patients (CAT ≥ 10 and LSA < 60) showed lower body mass index (BMI) and percent predicted forced expiratory volume in 1 s (%FEV_1_) than the other 3 groups. The prevalence of gastroesophageal reflex disease (GERD) was higher in symptomatic and physically active patients (CAT ≥ 10 and LSA ≥ 60) than less symptomatic and physically active patients (CAT < 10 and LSA ≥ 60) whereas the prevalence of allergic rhinitis, hypertension, ischemic heart disease, and diabetes mellitus did not differ between the 4 groups.

**Table 2:** Clinical and physiological comparisons between subgroups defined based on symptoms and physical activity questionnaires.

	Less symptomatic (CAT < 10)		Symptomatic (CAT ≥ 10)	
	LSA > 60 (n = 82)	LSA ≤ 60 (n = 11)	LSA > 60 (n = 73)	LSA ≤ 60 (n = 27)
Age	72.1 ± 6.4	74.0 ± 6.9	71.9 ± 8.4	74.3 ± 9.6
Male, n (%)	79 (96%)	9 (82%)	67 (92%)	25 (93%)
Pack-years	60.0 ± 30.7	76.1 ± 39.2	57.8 ± 28.7	60.9 ± 33.7
Height, cm	166.7 ± 6.5	160.2 ± 8.4	164.9 ± 6.9	163.3 ± 8.3
BMI, kg/m^2^	23.8 ± 3.2	23.3 ± 3.0	23.6 ± 3.6	20.0 ± 4.2*†‡
No. exacerbations in a previous year, 0/1/ ≥ 2	68/13/1	11/0/0	50/19/4	19/5/3
FEV_1_, % predicted	69.1 ± 20.4	71.3 ± 16.1	60.4 ± 23.3	45.4 ± 19.3*†‡
FVC, % predicted	95.0 ± 20.5	90.4 ± 6.4	87.9 ± 2.5	75.2 ± 4.1*‡
GERD	7 (9%)	2 (18%)	21 (29%) *	3 (11%)
Allergic rhinitis	10 (12.1%)	0 (0%)	12 (16.4%)	1 (3.7%)
Hypertension	41 (50%)	5 (45%)	48 (66%)	11 (41%)
IHD	13 (16%)	1 (9%)	12 (16%)	1 (4%)
DM	9 (11%)	2 (18%)	12 (16%)	2 (7%)
LAMA use, n (%)	43 (52%)	7 (64%)	46 (63%)	23 (85%) *
LABA use, n (%)	46 (56%)	5 (45%)	52 (71%)	20 (74%)
ICS use, n (%)	32 (39%)	2 (18%)	37 (51%)	11 (41%)

Data are expressed as mean ± SD and n (%). Based on scores of COPD assessment test (CAT) and Life-Space Assessment (LSA) questionnaires, patients were divided into 4 groups. IHD = ischemic heart disease, GERD = gastroesophageal reflex disease, DM = diabetes mellitus, LAMA = long-acting muscarinic antagonist, LABA = long-acting beta agonist, ICS = inhaled corticosteroid. * p < 0.05, †p < 0.05, and ‡ p < 0.05 compared to patients with CAT < 10 and LSA > 60, to those with CAT < 10 and LSA ≤ 60, and to those with CAT ≥ 10 and LSA > 60, respectively, based on the Tukey’s method for continuous variables and Fisher’s exact tests with Bonferroni correction for categorical variables.

### Pulmonary and extrapulmonary CT findings in symptomatic and physically inactive patients

As shown in Table 3, the symptomatic and physically inactive patients (CAT ≥ 10 and LSA < 60) exhibited higher low attenuation volume percentage on inspiratory CT (In-LAV_950_%, a marker for emphysema), low attenuation volume percentage on expiratory CT (Ex-LAV_856_%, a marker for air-trapping) and the volume percentage of non-emphysematous air-trapping regions reflecting small airway dysfunction (SAD%) than the other 3 groups. In contrast, the less symptomatic and physically inactive patients (CAT < 10 and LSA < 60) tended to exhibit lower In-LAV_950_%, Ex-LAV_856_%, and SAD% compared to the other groups. The airway dimension expressed as wall area percent (WA%) of subsegmental airways did not differ between the 4 groups.

**Table 3:** Comparisons of computed tomographic findings between subgroups defined based on symptoms and physical activity questionnaires.

	Less symptomatic (CAT < 10)		Symptomatic (CAT ≥ 10)	
	LSA > 60 (n = 82)	LSA ≤ 60 (n = 11)	LSA > 60 (n = 73)	LSA ≤ 60 (n = 27)
In-LAV_950_%, %	13.0 ± 11.6	8.8 ± 9.2	15.9 ± 12.3†	23.5 ± 14.2*†‡
Ex-LAV_856_%, %	42.0 ± 18.1	29.5 ± 14.8	46.0 ± 18.5	60.1 ± 11.7*†‡
SAD%, %	26.9 ± 11.5	18.8 ± 10.0	28.4 ± 11.2	35.8 ± 11.0*†‡
WA%, %	63.9 ± 3.6	63.2 ± 2.4	65.1 ± 3.6	63.9 ± 2.7
CLE, n (%)	40 (49%)	5 (45%)	43 (59%)	21 (78%)
PSE, n (%)	42 (51%)	4 (36%)	28 (38%)	16 (59%)
TLC_CT_, % predicted	94.9 ± 14.0	86.8 ± 14.0	95.9 ± 14.1	93.7 ± 14.8
FRC_CT_, % predicted	111.0 ± 25.8	88.6 ± 15.7	115.0 ± 30.0†	125.0 ± 23.4†
PM index, cm^2^ /m^2^	10.5 ± 2.4	9.1 ± 1.2	10.3 ± 3.0	8.4 ± 2.7*‡
SAT index, cm^2^ /m^2^	10.1 ± 3.9	10.6 ± 3.0	10.6 ± 4.6	6.7 ± 3.9*†‡
ESM index, cm^2^ /m^2^	12.0 ± 2.5	11.5 ± 2.1	12.1 ± 3.0	10.5 ± 2.5‡
BMD, HU	159.2 ± 43.6	101.0 ± 36.7*	147.0 ± 48.6†	144.0 ± 49.7†
Agatston score > 400, n (%)	19 (23%)	3 (27%)	22 (30%)	8 (30%)

Data are expressed as mean ± SD and n (%). Based on scores of COPD assessment test (CAT) and Life-Space Assessment (LSA) questionnaires, patients were divided into 4 groups. In-LAV_950_% = low attenuation volume percentage on inspiratory CT. Ex-LAV_856_% = low attenuation volume percentage on expiratory CT (air-trapping index). SAD% = non-emphysematous air-trapping percentage (small airway dysfunction) on inspiratory/expiratory CT. WA% = wall area percent. CLE = centrilobular emphysema. PSE = paraseptal emphysema. TLC_CT_ = total lung volume on inspiratory CT adjusted by reference total lung capacity value. FRC_CT_ = total lung volume on expiratory CT adjusted by reference functional residual capacity value. PM index = cross-sectional area of pectoralis muscle that was normalized by height. SAT index = cross-sectional area of subcutaneous adipose tissue adjacent to pectoralis muscle that was normalized by height. ESM index = cross-sectional area of erector spinae muscle that was normalized by height. BMD = thoracic vertebral bone mineral density. Agatston score = coronary artery calcification score. * p < 0.05, †p < 0.05, and ‡ p < 0.05 compared to patients with CAT < 10 and LSA > 60, to those with CAT < 10 and LSA ≤ 60, and to those with CAT ≥ 10 and LSA > 60, respectively, based on the Tukey’s method for continuous variables and Fisher’s exact tests with Bonferroni correction for categorical variables.

With respect to extrapulmonary CT indices, the symptomatic and physically inactive patients (CAT ≥ 10 and LSA < 60) exhibited smaller cross-sectional areas of pectoralis and erector spinae muscles and subcutaneous adipose tissue adjacent to pectoralis muscle that were normalized by height (PM index, ESM index, and SAT index respectively) compared to the symptomatic and physically active patients (CAT ≥ 10 and LSA ≥ 60). In contrast, no difference in PM index, ESM index, or SAT index was found between the less symptomatic physically inactive patients (CAT < 10 and LSA < 60) and the less symptomatic and physically active patients (CAT < 10 and LSA ≥ 60). BMD on thoracic vertebra expressed as mean CT values was lower in the less symptomatic physically inactive patients. The prevalence of coronary artery calcification defined as the Agatston score > 400 did not differ between the 4 groups.

### Multivariable analyses to explore relative associations of pulmonary and extrapulmonary CT findings with impaired physical activity (LSA < 60) in symptomatic patients

**Figure 2:**
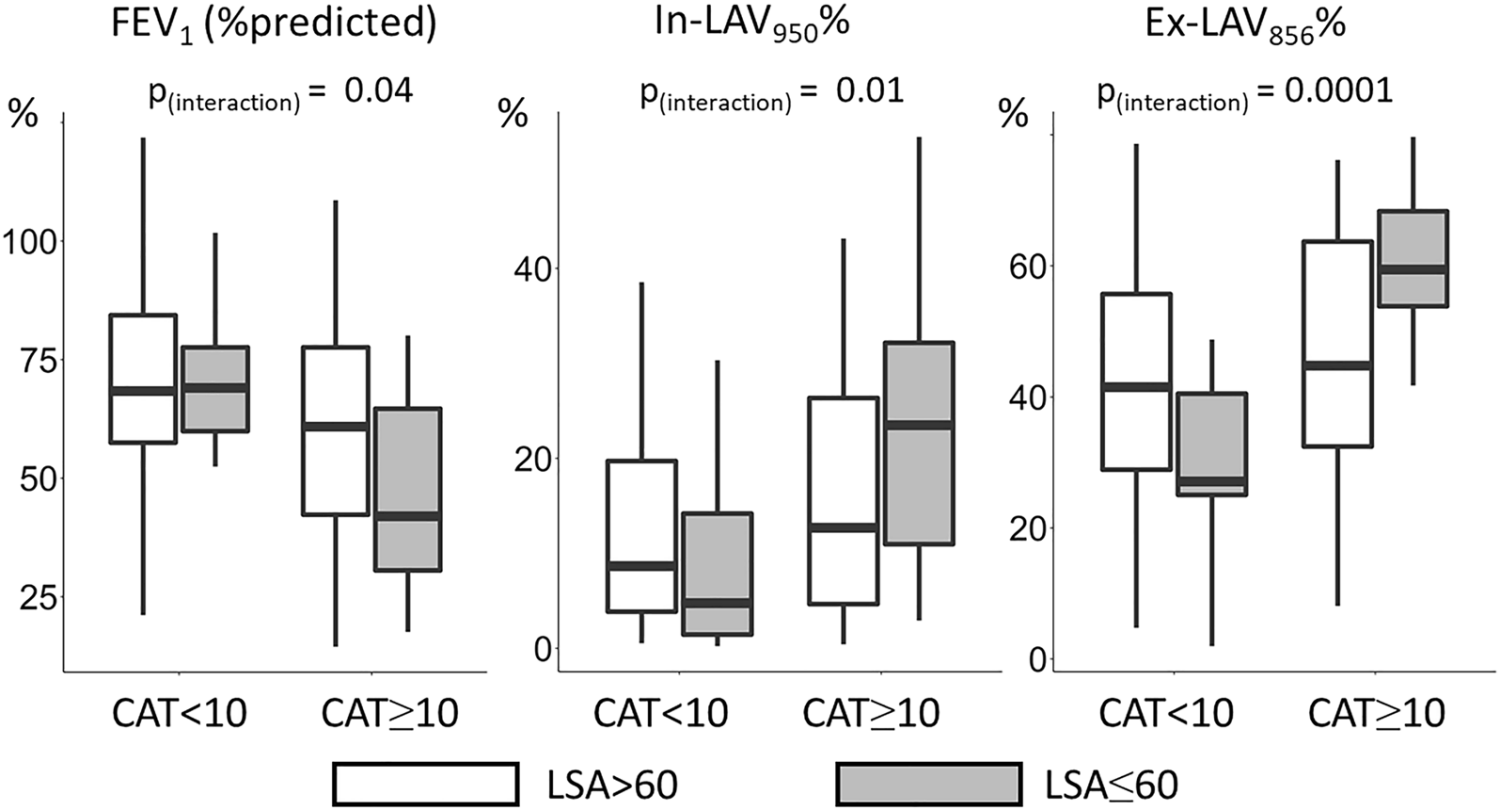
Airflow limitation, emphysema and air-trapping in subgroups defined based on symptoms and physical activity. Based on scores of COPD assessment test (CAT) and Life-Space Assessment (LSA), patients with COPD were classified into 4 groups. There were significant interactions between CAT and LSA on FEV_1_, In-LAV_950_%, and Ex-LAV_856_% (*p* = 0.04, 0.01 and 0.0001, respectively).

As shown in Fig. 2, there were significant interactions between CAT and LSA on FEV_1_, In-LAV_950_%, and EX-LAV_856_% (p = 0.04, 0.01 and 0.0001, respectively). Thus, further examinations focused on the associations of pulmonary and extrapulmonary CT findings with the low LSA score (LSA < 60) in symptomatic patients (CAT ≥ 10). As shown in Table 4, multivariable logistic regression models (Model 1 and 2) showed significant associations between PM index and the low LSA score (odds ratio [95% CI] = 2.68 [1.31, 6.11]) and between SAT index and the low LSA score (odds ratio [95% CI] = 3.27 [1.61, 7.60]) independent of In-LAV_950_%, BMD, coronary artery calcification (the Agatston score > 400), and the demographic factors. Moreover, in Model 3, 1-SD-increment of Ex-LAV_856_% and 1-SD-decrement of PM index were independently associated with the low LSA (odds ratio [95% CI] = 2.26 [1.22, 4.59] and 2.31 [1.09, 5.34], respectively). In Model 4, 1-SD-increment of Ex-LAV_856_% and 1-SD-decrement of SAT index were independently associated with the low LSA (odds ratio [95% CI] = 2.31 [1.25, 4.68] and 3.01 [1.45, 7.16], respectively). In Model 5, 1-SD-increment of SAD% and 1-SD-decrement of PM index were independently associated with the low LSA (odds ratio [95% CI] = 2.10 [1.18, 4.05] and 3.08 [1.50, 7.11], respectively). In Model 6, 1-SD-increment of SAD% and 1-SD-decrement of SAT index were independently associated with the low LSA (odds ratio [95% CI] = 1.98 [1.11, 3.84] and 3.53 [1.72, 8.28], respectively).

**Table 4:** Multivariable logistic models to explore associations of CT findings with impaired physical activity in symptomatic patients with COPD (n = 100).

	Model 1	Model 2	Model 3	Model 4	Model 5	Model 6
In-LAV_950_%, per 1-SD-increment	1.50 [0.89, 2.62]	1.54 [0.90, 2.7]	–	–	–	–
Ex-LAV_856_%, per 1-SD-increment	–	–	2.26* [1.22, 4.59]	2.31* [1.25, 4.68]	–	–
SAD%, per 1-SD-increment	–	–	–	–	2.10* [1.18, 4.05]	1.98* [1.11, 3.84]
PM index, per 1-SD-decrement	2.68* [1.31, 6.11]	-	2.31* [1.09, 5.34]	–	3.08* [1.50, 7.11]	–
SAT index, per 1-SD-decrement	–	3.27* [1.61, 7.60]	–	3.01* [1.45, 7.16]	–	3.53* [1.72, 8.28]
BMD, per 1-SD-decrement	0.79 [0.42, 1.46]	1.00 [0.53, 1.94]	0.76 [0.40, 1.42]	0.87 [0.44, 1.74]	0.67 [0.36, 1.25]	0.87 [0.45, 1.69]
Agatston score > 400, yes/no	0.65 [0.21, 1.92]	0.51 [0.14, 1.60]	0.55 [0.16, 1.68]	0.39 [0.10, 1.32]	0.61 [0.18, 1.83]	0.44 [0.12, 1.44]

Each model was adjusted by age, sex, smoking pack-years, and institution. Impaired physical activity was defined as Life-Space Assessment (LSA) score < 60. All patients met COPD assessment test (CAT) ≥ 10. Values indicate estimate [95% confidence interval]. “- “ indicates no inclusion for a given model. * p < 0.05. In-LAV_950_% = low attenuation volume percentage on inspiratory CT. Ex-LAV_856_% = low attenuation volume percentage on expiratory CT (air-trapping index). SAD% = non-emphysematous air-trapping percentage (small airway dysfunction) on inspiratory/expiratory CT. PM index = cross-sectional area of pectoralis muscle that was normalized by height. SAT index = cross-sectional area of subcutaneous adipose tissue adjacent to pectoralis muscle that was normalized by height. BMD = Thoracic vertebral bone mineral density. Agatston score = coronary artery calcification score.

**Figure 3:**
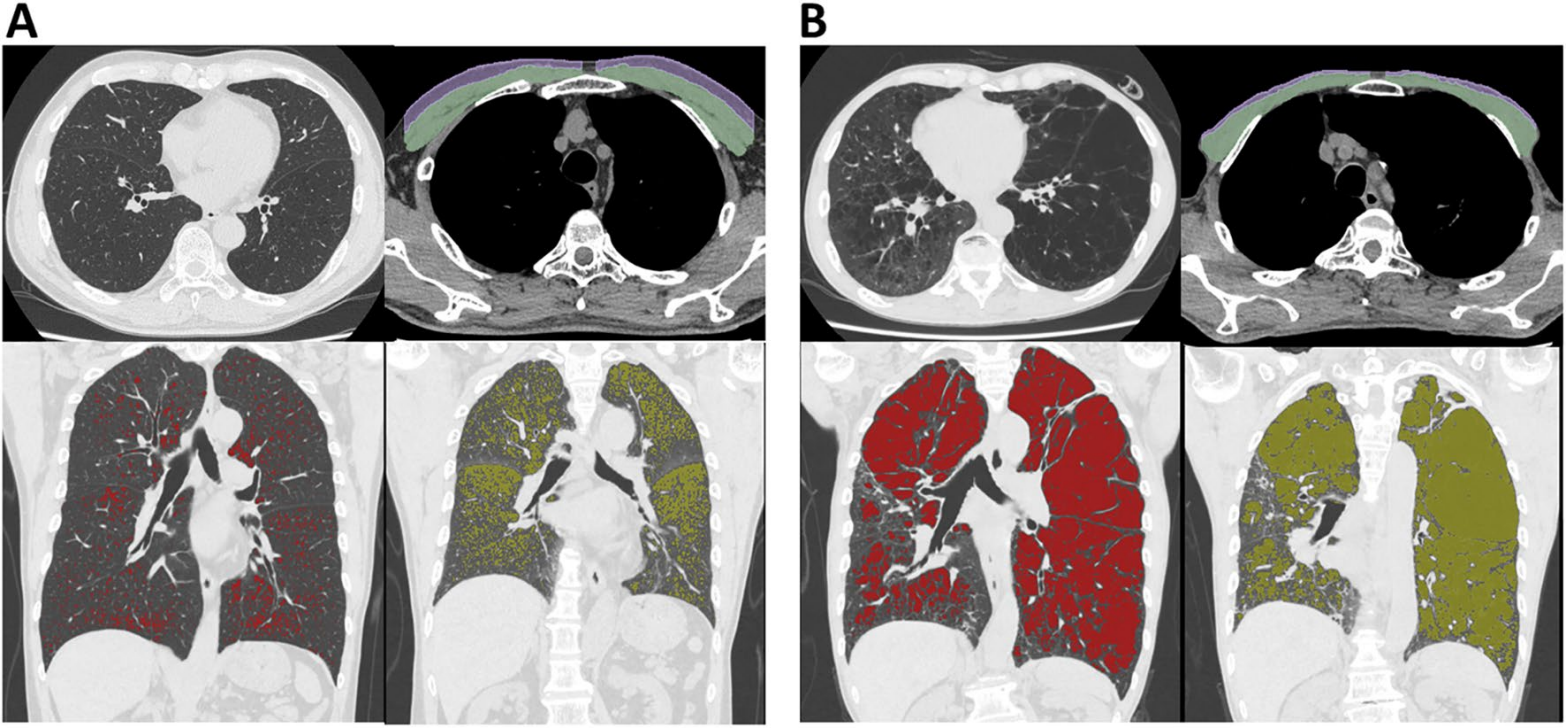
Examples of pulmonary and extrapulmonary CT findings in symptomatic patients with and without physical inactivity. (**A**) Inspiratory and expiratory CT in symptomatic patient with COPD and preserved physical activity (COPD assessment test [CAT] = 20 and Life-Space assessment [LSA] = 96). (**B**) CT in symptomatic patient with COPD and impaired physical activity (CAT = 21 and LSA = 45). Green and purple segmentation indicate pectoralis muscles and subcutaneous adipose tissues. Red and yellow regions indicate emphysema on inspiratory CT and air-trapping on expiratory CT. Of note, pectoralis muscle and adjacent subcutaneous adipose tissue were smaller, and emphysema and air-trapping were more severe in the patient with lower LSA (B) than in the patient with higher LSA (A).

Figure 3 shows representative CT images in symptomatic patients with preserved and impaired physical activity (A: CAT = 20 and LSA = 96, and B: CAT = 21 and LSA = 45, respectively). The patient with impaired physical activity exhibited smaller cross-sectional areas of pectoralis muscle and adjacent subcutaneous adipose tissue and increased emphysematous regions on inspiratory CT and increased air-trapping on expiratory CT compared to the patient with preserved physical activity.

## Discussion

This study showed that the symptomatic and physically inactive state (CAT ≥ 10 and LSA < 60) was found in 14% patients with COPD and associated with increases in In-LAV_950_%_,_ Ex-LAV_856_%, and SAD% in the lungs and decreases in PM index, SAT index, and ESM index outside the lungs. Additionally, the study demonstrated the interaction between the symptoms (CAT ≥ 10) and physical inactivity (LSA ≤ 60) on FEV_1_, In-LAV_950_%_,_ and Ex-LAV_856_%. Moreover, the multivariable analysis showed that increase in Ex-LAV_856_% and decrease in PM index and SAT index were independently associated with the low LSA in symptomatic patients (CAT ≥ 10). Although papers have shown that lung hyperinflation due to air-trapping and muscle wasting are associated with impaired physical activity^25,26^, to best of knowledge, this study is the first to comprehensively examine pulmonary and extrapulmonary abnormalities using CT and to show the independent associations of increased air-trapping and decreased skeletal muscle and subcutaneous adipose tissue quantity with impaired physical activity in symptomatic patients with COPD. The findings are clinically relevant as they suggest that both relieving air-trapping as well as restoring muscle wasting and body composition should be considered to improve physical activity in symptomatic patients.

The significant interactions between the symptoms and physical inactivity on pulmonary function and structures are important findings in this study. In less symptomatic patients with COPD (CAT < 10), the impaired physical activity (LSA ≤ 60) was not associated with worsening of FEV_1_, emphysematous changes, and air-trapping in the lungs. This might be because physical activity can be affected dominantly by social and environmental factors, mental status and working status^25^ in these patients. In contrast, in symptomatic patients with COPD (CAT ≥ 10), the low LSA was associated with lower FEV_1_ and higher air-trapping. These findings suggest the importance of simultaneous evaluation of symptoms when interpreting the physical activity in patients with COPD.

In the multivariable models of symptomatic patients (Table 4), the associations of 1-SD increments of Ex-LAV_856_% and SAD% with the low LSA (odds ratio for Ex-LAV_856_% = 2.26 and 2.31 in Model 3 and Model 4; odds ratio for SAD% = 2.10 and 1.98 in Model 5 and Model 6) were greater than that of 1-SD increment of In-LAV_950_% (odds ratio = 1.50 and 1.54 in Model 1 and 2). Previous studies have shown that hyperinflation, especially dynamic hyperinflation on exertion, as well as emphysema are associated with physical inactivity^4,25,26^. Because Ex-LAV_856_% is a CT index of air-trapping mainly influenced by emphysema and small airway disease^27^ and SAD% is a CT index of non-emphysematous air-trapping presumably induced by small airway disease^28,29^, our data extend the literature by suggesting that air-trapping induced mainly by small airway disease is associated with physical inactivity in symptomatic COPD.

In addition to lower PM index, lower SAT index was associated with impaired physical activity (LSA < 60) in the symptomatic patients. This finding is consistent with previous reports showing that lower SAT was associated with higher mortality^20^ and that higher mortality was associated with impaired physical activity in patients with COPD^7^. Meanwhile, Martinez CH et al.^30^ showed that higher SAT and lower PM index were associated with lower handgrip strength that was a risk factor of COPD exacerbations. Since both underweight and extreme obesity (BMI ≥ 40) cause poor prognosis in patients with COPD^31^, the impact of SAT on clinical outcomes might differ between patients with underweight and those with obesity. In this study, the prevalence of obesity (BMI ≥ 30) (3%) was very low, which is consistent with a reported feature of Asian patients with COPD^32^. Therefore, we postulate that combined reductions in skeletal muscle and subcutaneous adipose tissues could be a distinct extrapulmonary manifestation associated with symptoms and physical inactivity in non-obese patients with COPD.

The coronary artery calcification (the Agatston score > 400) was not associated with the low LSA. Physical inactivity is associated with the progression of coronary artery calcification in adult subjects in the population-based cohort in the United States^33^. Moreover, a longitudinal study of patients with COPD showed that coronary artery calcium score was associated with increased mortality^22^. The lack of association between coronary artery calcification and low LSA score in this study might be because the prevalence and prognostic impacts of cardiovascular disease are lower in Japanese patients with COPD than in those in the Western countries^32^.

The low LSA was associated with lower BMD in less symptomatic patients, but not in symptomatic patients. Furthermore, Ex-LAV_856_% did not differ between less symptomatic (CAT < 10) and symptomatic (CAT ≥ 10) patients with LSA > 60. Although higher CAT has been shown to be associated with severe airflow limitation and emphysema^34–36^, this finding suggests that symptoms (CAT ≥ 10) could not be directly associated with air-trapping in patients with the relatively preserved physical activity. Together with the observed interactions between CAT and LSA on pulmonary pathophysiological indices, we believe that the combined use of CAT and LSA questionnaires would be more useful to identify patients with structural and physiological abnormalities than an individual use of either CAT or LSA questionnaire.

 The importance of personalized management according to distinct clinical features and treatable traits is increasingly recognized^37^. Since increased air-trapping and combined decreases in skeletal muscle mass and subcutaneous adipose tissue are independently associated with the symptomatic and physically inactive state (CAT ≥ 10 and LSA < 60), a combinational approach including not only treatments with bronchodilators, pulmonary rehabilitation, and lung volume reduction procedure to relieve air-trapping but also nutritional support and rehabilitative exercise training to restore skeletal muscle quantity and body composition should be provided for improving physical activity in these patients^11,38^. For the symptomatic and physically active patients (CAT ≥ 10 and LSA ≥ 60), in addition to pharmacological treatment, self-management education to increase motivation to maintain physical activity would be important. Moreover, the higher prevalence of GERD in these patients (CAT ≥ 10 and LSA ≥ 60) is consistent with a previous report on an association between GERD and chronic bronchitis symptoms^39^. GERD increases a risk of exacerbation and hospitalization in patients with COPD^40^, but is treatable by medication. Much attention should be paid to the possibility of GERD in this group.

This study has several limitations. First, the sample size was relatively small. However, we enrolled diverse subjects from two institutions (clinic and university hospital) whose clinical settings are different. This increases the generalizability of the present findings. Second, physical activity was assessed using the LSA questionnaire. Since direct evaluation using an accelerometer is widely used to evaluate physical activity^7^, future studies should investigate whether such a direct evaluation of physical activity combined with CAT score would reproduce the present findings.

In conclusion, CT assessments in and outside the lungs demonstrated that air-trapping and decrease in skeletal muscle and subcutaneous adipose tissue quantity were independently associated with physical inactivity in highly symptomatic patients with COPD. These confirm the importance of incorporating both pulmonary and extrapulmonary CT findings to achieve a more personalized management of COPD and suggest that both relief in air-trapping in the lungs and restoring body composition might be targets for improving physical activity in patients with COPD.

## Methods

### Ethics

This study used the baseline data of the Kyoto-Himeji Cohort that is an ongoing prospective observational study conducted at the Kyoto University hospital and Terada clinic, in Japan. The study was conducted in accordance with the Declaration of Helsinki, approved by the Ethics Committee of Kyoto University (approval No. C1311, approval date November 8, 2017), and registered with the University Hospital Medical Information Network (UMIN000028387). All participants provided written informed consent.

### Study subjects

Stable smokers at age ≥ 40 years with a history of ≥ 10 pack-years were enrolled from 2018 to 2020 and underwent spirometry and a pair of inspiratory and expiratory chest CT during exacerbation-free period. The exclusion criteria were as follows: (1) inappropriate breath holding during CT scans, (2) a history of other respiratory diseases such as interstitial lung disease and lung cancer, (3) current primary diagnosis of asthma, (4) the incompleteness of the questionnaire, and (5) insufficient cognitive function judged by physicians. A diagnosis of COPD was based on GOLD criteria^1^. Spirometry was performed after inhalation of bronchodilator using Chestac-8900 (Chest M.I. Inc., Tokyo, Japan) in the Kyoto University and Microspiro HI-302U (Nihon Kohden, Tokyo, Japan) in the Terada Clinic, and the predicted forced vital capacity (FVC) and predicted FEV_1_ were calculated with the LMS method^41^.

### Questionnaires

Respiratory symptoms and physical activity were evaluated using self-administered questionnaires including the modified Medical Research Council (mMRC), CAT and LSA. CAT score ≥ 10 indicated substantial symptoms^1,42^. Number of exacerbations defined as the use of oral corticosteroids or antibiotics or the need for hospitalization due to worsening of respiratory symptoms was recorded in a previous year. The LSA questionnaire consisted of 5 questions regarding life-space mobility, including frequency and independence during the 4 weeks before the evaluation. The score ranged from 0 to 120; a higher score indicated a more active status ^24,43^. Scores < 60 reflected restricted physical activity and social isolation ^44^.

### CT acquisition

CT images with 512 × 512 matrix and 1 mm slice thickness for the entire lungs were obtained at full inspiration and end-tidal expiration using an Aquilion Precision scanner at Kyoto University and an Aquilion Lightning scanner at Terada Clinic (Canon Medical Systems, Otawara, Japan). Images reconstructed with the soft (FC13) kernel were used to quantify lung density and extrapulmonary features, whereas images reconstructed with the sharp (FC51) kernel were used to quantify airway dimension, respectively^45^. The scanning conditions were 120 kVp, 0.5-s exposure time, and auto-exposure control.

### CT analyses of lung and airway

Using a SYNAPSE VINCENT software (FUJIFILM, Tokyo, Japan), the volume percentage of emphysema regions defined as low attenuation voxels < -950 HU on inspiratory CT (In-LAV_950_%)^45^ and the volume percentage of air-trapping regions defined as low attenuation voxels < -856 HU on expiratory CT (Ex-LAV_856_%)^16^ were calculated for the entire lung. Additionally, expiratory CT was non-rigidly registered to inspiratory CT and the volume percentage of non-emphysematous air-trapping regions reflecting small airway dysfunction^28,29^, defined as voxels ≥  − 950 HU on inspiratory CT and < -856 HU on expiratory CT (SAD%), was calculated. Centrilobular emphysema (CLE) and paraseptal emphysema (PSE) were also visually identified based on the Fleischner Society classification system^46^. In this study, CLE refers to moderate to advanced CLE and PSE refers to substantial PSE. Lung volumes on inspiratory and expiratory CT were adjusted by reference total lung capacity and functional residual volume values, respectively (TLC_CT_ % predicted and FRC_CT_ %precited) using previously reported equations^47^. To evaluate the airway dimension, the lumen, wall area, and wall area percent (WA%) of the right apical and posterior basal subsegmental bronchus were measured and averaged^45^.

### CT analyses of extrapulmonary features

Pectoralis muscle, erector spinae muscle, subcutaneous adipose tissue, BMD on thoracic vertebra and the Agatston score on inspiratory CT were quantitatively evaluated using Image J (Fiji) software^48^ and custom-made scripts implemented in Python. On the first axial slice above the aortic arch, the left and right pectoralis muscles (major and minor) were manually segmented from regions with CT values ranging between − 29 and + 150 HU as previously reported^18,49^. Then, subcutaneous adipose tissue was automatically identified as regions located between the pectoralis muscles and the skin surface on the same axial slice^20^. Additionally, on a single axial slice at the level of the lower margin of the 12^th^ thoracic vertebra, the left and right erector spinae muscles were segmented manually, and their area were summed^19,21^. Cross-sectional areas of pectoralis muscle, erector spinae muscle, and subcutaneous adipose tissue were normalized by dividing them by squared height, which were termed PM index, ESM index, and SAT index, respectively.

BMD was evaluated by averaging mean CT value at the 4^th^, 7^th^, and 10^th^ thoracic vertebral^17^. The elliptical region of interest was manually placed within the body of vertebral as large as possible at each mid-vertebral slice, and mean CT value was measured for T4, T7, and T10. To obtain the Agatston score^50^, areas of coronary calcium with CT density of ≥ 130 HU and ≥ 1mm^2^ along with coronary artery were measured in each axial slice, multiplied by weighting factor defined according to their maximal CT density, summed up and standardized by multiplying the score by the slice thickness (1 mm in this study) and dividing by 3 mm because the original Agatston score was based on 3 mm slice-thickness as previously reported^51^. The Agatston score ≥ 400 was considered as the presence of coronary artery calcification in this study.

### Statistical analysis

Statistical analysis was performed using JMP Pro 14 (SAS institute, Cary, NC, USA) and R statistical software version 4.0.1^52^. Data are expressed as the mean and standard deviation (SD) unless indicated. Continuous variables were compared using one-way ANOVA followed by Tukey’s multiple comparison method. Square root transformation was performed when necessary. For categorical variables, Fisher's exact test was used and multiple comparisons were adjusted by the Bonferroni correction. Interactions between LSA and CAT on FEV_1_, In-LAV_950_%, and Ex-LAV_856_% were assessed using multivariable linear regression model. Furthermore, multivariable linear regression models were constructed to explore associations between low LSA and each parameter in symptomatic patients with COPD (CAT ≥ 10). P values < 0.05 were considered statistically significant.

## Availability of data and material

The datasets used and analyzed during the current study are available from the corresponding author on reasonable request.
